# Dynamic visualization of brain pulsations using amplified MRI: methodology and applications

**DOI:** 10.1098/rsfs.2024.0049

**Published:** 2025-04-04

**Authors:** Haribalan Kumar, Mehmet Kurt, Josh McGeown, Paul Condron, Jet Wright, Gonzalo Maso Talou, Joonsung Lee, Itamar Terem, Helen Danesh-Meyer, Eryn Kwon, Samantha Holdsworth

**Affiliations:** ^1^ GE HealthCare, Tairāwhiti-Gisborne, New Zealand; ^2^ Department of Mechanical Engineering, University of Washington, Seattle, WA, USA; ^3^ Mātai Medical Research Institute, Tairāwhiti-Gisborne, New Zealand; ^4^ Department of Anatomy and Medical Imaging, Faculty of Medical and Health Sciences and Centre for Brain Research, University of Auckland, Auckland, New Zealand; ^5^ Auckland Bioengineering Institute, University of Auckland, Auckland, New Zealand; ^6^ GE HealthCare, Seoul, South Korea; ^7^ Department of Electrical Engineering, Stanford University, Stanford, CA, USA; ^8^ Department of Ophthalmology, University of Auckland, Auckland, New Zealand; ^9^ Vision Research Foundation, Auckland, New Zealand

**Keywords:** magnetic resonance imaging, dynamic imaging, intracranial pulsatility, computer vision, dynamic mode decomposition, amplified MRI (aMRI), cine MRI, brain imaging, pulsatile brain motion

## Abstract

Brain pulsatility offers a compelling application in the study of cerebral biomechanics, particularly for mild traumatic brain injury (mTBI) and elevated intracranial pressure (ICP). In this study, we used amplified MRI to quantify brain tissue pulsations. Dynamic mode decomposition (DMD) processing was then applied to provide a spatio-temporal analysis of motion. Four distinct use cases were examined: (i) resting versus exertion-induced heart rate changes, (ii) pre- and post-lumbar puncture (LP), (iii) baseline versus post-brain injury, and (iv) a test–retest case. Results demonstrate that brain tissue motion varies significantly across conditions, with DMD revealing distinct modes and frequencies corresponding to physiological changes. Notably, mTBI showed an increase in pulsatile motion post-injury, while elevated ICP exhibited altered pulsatility patterns post-LP, indicating a potential biomarker for injury and pressure-related changes. This approach offers new insights into physiological and pathological brain pulsatility; however, the study’s limited sample size, reliance on retrospective gating and assumptions regarding pulsatile motion highlight the need for larger and more diverse cohorts to confirm these findings. Despite these limitations, our results suggest our dynamical analysis approach could become a valuable tool for assessing intracranial dynamics, with applications in clinical diagnostics and research on neurovascular and neurological conditions.

## 1. Introduction

Brain pulsatility can be assessed using various imaging modalities, each offering unique advantages. MRI provides very high spatial resolution of subcortical structures, white and grey matter and brain parenchyma. However, conventional anatomical MRI lacks sufficient temporal resolution compared with other imaging techniques, such as transcranial Doppler ultrasound and Doppler optical coherence tomography, which are commonly used to capture dynamic brain tissue pulsations in real time. For example, transcranial Doppler ultrasound non-invasively measures cerebral blood flow velocity through an external electrical probe placed near the cranium, making it effective for tracking rapid pulsatile changes in blood flow [1–4].

Multiple MRI methods exist for imaging brain pulsatility, each contributing unique strengths in assessing the dynamic motion of brain tissues and fluids. For example, phase-contrast MRI (PC-MRI) [5,6] is widely used to measure cerebrospinal fluid (CSF) and blood flow velocities across the cardiac cycle, particularly in localized regions such as major cerebral arteries and the cerebral aqueduct. PC-MRI is also used to capture brain parenchyma motion, with applications in diagnosing conditions like Chiari malformation [7,8].

Tagged MRI techniques, initially developed for cardiac imaging, have also been adapted to track brain tissue displacement by using motion-sensitizing gradients or tagging patterns on the tissue, effectively capturing subtle pulsatile movements within the brain parenchyma [9,10]. These techniques have been applied to measure brain deformation in response to mild head acceleration, simulating conditions seen in mild traumatic brain injury (mTBI) [11]. Closely related to tagged MRI, displacement encoding with stimulated echoes (DENSE)-MRI [12] directly encodes tissue displacement in the phase of the signal, providing more quantitative displacement data—with applications in conditions like Chiari malformation [13].

Cine balanced steady-state free precession (bSSFP) imaging [14] provides high tissue contrast, allowing researchers to create dynamic ‘cine loops’ of brain tissue motion across the cardiac cycle, which have been useful in clinical applications such as evaluating subarachnoid spaces [15]. Real-time bSSFP [16] offers continuous imaging without gating, allowing for dynamic tracking of motion, such as condyle movement during voluntary mouth opening in speech studies. This capability shows promise for clinical scenarios requiring real-time/immediate visualization of brain motion.

A more recent innovation, amplified MRI (aMRI) [17–21], introduces the advantage of enhancing small, otherwise imperceptible brain tissue displacements by applying motion magnification algorithms. This technique magnifies subtle movements and fluid dynamics in the brain, providing valuable insights into neurological conditions like Chiari malformation [17] and neurodegenerative diseases [19] and acute haemorrhagic stroke [22]. aMRI can capture three-dimensional (3D) brain motion with a spatial resolution of approximately 1.2 mm in under 2 min of scan time, making it highly efficient and accessible for clinical settings.

Despite the progress in MRI techniques for assessing brain pulsatility, the application of these methods, especially aMRI, to intracranial pulsatility measurement is still relatively new. Much remains to be studied to establish normative ranges of pulsatility across different age groups and in healthy versus pathological states. In our experience, interpreting pulsatile motion in amplified images has proven challenging due to the lack of standardized terminology to describe such subtle, dynamic changes. Previous studies have often relied on qualitative descriptions, which limits precise reporting and comparability. In this work, we present a new method for quantifying and reporting intracranial pulsatility using aMRI, aiming to contribute a more standardized approach to this emerging field.

Imaging pulsatility through MRI-based approaches offers unique advantages in assessing pathological changes in the brain, such as alterations in intracranial pressure (ICP), cerebral oedema, vascular flow abnormalities and CSF blockages. Such techniques also enable the measurement of displacement or motion, which can then be converted to strain estimates. Second, there is a fundamental, causal link between tissue motion and stiffness or compliance, meaning that estimating pulsatility through motion extraction provides valuable insights into this relationship.

When dealing with intracranial pulsatility, it is important to distinguish between motion that is visible at the resolution of the image and motion that is subvoxel, or not and often not directly perceptible. Our focus is on the latter, where subtle, subvoxel intensity fluctuations caused by arterial pulsatility are synchronized with the cardiac cycle but remain imperceptible in standard cine MRI images. This approach to estimating motion from regular cine images is relatively new in the context of intracranial pulsatility, though subpixel motion estimation is well established in other fields, such as machine vision and image processing. For instance, techniques like digital volume correlation and 3D point tracking are used to detect vibrations in structures by analysing variations in phase within the frequency domain.

This article aims to demonstrate intracranial pulsatility using aMRI. Our hypothesis is that any changes in pulsatility will be detectable, with two image acquisition points provided for each case as described below. Traditionally, aMRI is analysed by looking into difference maps of the amplified images and using quantitative displacement patterns within the frequency band, and our perspective here is whether dynamic mode decomposition (DMD) can capture the perceptual intensity variation patterns and be used as a simple, streamlined method for a quick analysis of aMRI data. To achieve this aim, the dynamics are analysed using DMD [23,24], a method that breaks down complex, changing motions into simple, repeating patterns in the spatio-temporal domain to help analyse subtle movements. We have selected three use cases: (i) exercise-induced elevation in heart rate (HR), (ii) pre- and post-lumbar puncture (LP) to assess changes in ICP, and (iii) comparison of pulsatility pre- and post-mTBI, with an additional test–retest use case for consistency. In each case, we hypothesize that brain pulsatility will be altered in response to the physiological stimulus. DMD will allow us to assess intrasubject changes in motion patterns by evaluating shifts in frequency and amplitude.

### 1.1. Use case 1: change in heart rate

Characterizing the normal patterns and ranges of brain pulsatility could help identify biomarkers for various neurological conditions, as deviations from the ‘healthy’ range may be indicative of underlying pathology. Within this context, a key question is how different HRs impact these pulsatility patterns. Changes in HR can influence the dynamics of cerebral blood flow and CSF movement, leading to alterations in brain physiology. As HR increases, the cardiac cycle shortens, which may alter the timing and intensity of blood and CSF flow within the brain. If the energy driving these flows does not increase proportionally with the HR, the amplitude of physiological brain motions may decrease. Hence, this use case explores changes in pulsatility in healthy volunteers arising due to changes in cardiac output reflected as changes in average HR.

### 1.2. Use case 2: change in intracranial pressure

Elevated ICP is associated with neuroimaging signs such as distended optical nerve sheath, tortuous optical nerve and stenosed venous sinus. Elevated ICP is further associated with decreased cerebral perfusion and potential ischaemic injury, as increased pressure within the cranial vault restricts blood flow to the brain tissue. The brain operates as a three-compartment system consisting of brain tissue, CSF and blood. When ICP rises, volume adjustments occur across these compartments to maintain homeostasis, impacting the distribution of blood and CSF. Prior studies have shown that brain motion is influenced by tissue compliance and that fluid pressure changes, such as those following an LP, can affect brain pulsatility. Building on this evidence, we hypothesize that changes in ICP will alter brain motion patterns, and that aMRI may be able to detect these subtle changes in pulsatility.

Specifically, we anticipate that aMRI can detect alterations in brain motion in response to changes in ICP. To test this, we selected two time points representing the pre- and post-LP states to assess changes in brain motion patterns associated with ICP reduction. Our analysis focuses on three aspects: (i) examining differences in pulsatile brain motion by comparing pre- and post-LP conditions across the first harmonic or wider frequency band, (ii) detecting any frequency shifts in brain motion between the pre- and post-LP states, and (iii) assessing regional variations in brain pulsatility following a decrease in ICP. We aim to demonstrate that a reduction in ICP produces measurable changes in both the amplitude and frequency of brain motion patterns, providing new insights into how ICP modulation affects brain dynamics.

### 1.3. Use case 3: change due to mild traumatic brain injury

In cases of mTBI, commonly referred to as concussion, rapid stretching and shearing forces damage the neuronal and vascular cytoskeletal structures [25,26]. These forces initiate a cascade of neurophysiological disruptions, including impaired vasoreactivity, altered CBF, subtle diffuse cerebral oedema and neuroinflammation due to blood–brain barrier disruption. Evaluating these subtle, injury-induced changes in humans remains challenging [27], as standard clinical imaging, which primarily focuses on anatomical alterations, often fails to detect the functional disruptions associated with mTBI.

Measuring brain pulsatility by estimating voxel-level motion may offer a novel, non-invasive approach to assess potential links between mTBI pathophysiology and observable changes in brain tissue properties, such as parenchymal stiffness or glymphatic system function. This approach provides an opportunity to capture mTBI-related disruptions in brain dynamics that may otherwise remain undetected with conventional imaging techniques.

## 2. Methods

### 2.1. Participants

With approval from the New Zealand Health and Disability Ethics Committee (reference numbers: 20/CEN/107 and 20/NTB/14) and informed consent, four participants were recruited for this study. aMRI data were acquired using a 3 T MRI machine (SIGNA Premier; General Electric Healthcare, Milwaukee, WI) with a 48-channel AIR™ coil. Four distinct use cases were examined as outlined as follows.

#### 2.1.1. Use case 1: resting and exertion

Imaging was performed at both a resting state and during exertion, induced by a grip-ring exercise performed at 80% of the participant’s maximum effort. This exercise achieved a nominal 30% increase in HR, with an average recorded HR of 68 bpm at rest and 83 bpm following exertion.

#### 2.1.2. Use case 2: changes in intracranial pressure

The effect of changes in ICP was assessed in a 50-year-old female patient, with imaging performed before and after a LP. Recorded opening pressures were 32 cm H_₂_O pre-LP and 23 cm H_₂_O post-LP. Imaging data were collected within 30 min of the LP procedure.

#### 2.1.3. Use case 3: pre- and post-mild traumatic brain injury

Imaging data were acquired from a 16-year-old male athlete before and after experiencing mTBI. The two imaging sessions were 12 days apart, with the post-mTBI scan occurring within 48 h of the injury.

#### 2.1.4. Use case 4: test–retest

Here, a test–retest assessment was conducted on a 41-year-old male with two imaging sessions conducted 10 min apart using the same imaging protocol.

## 3. Image acquisition and processing

**Figure 1.**
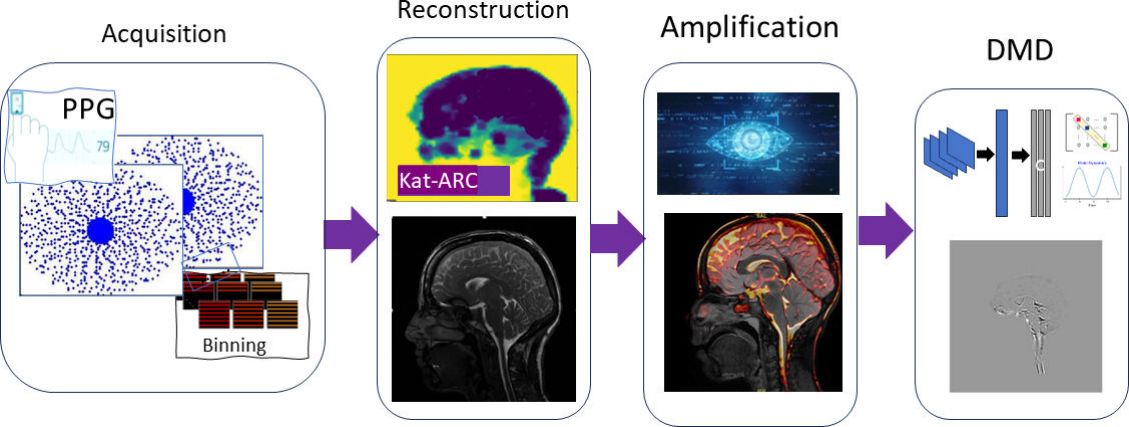
Workflow to extract motion from 3D cine dynamic images. The workflow consists of three stages: acquisition, reconstruction and motion estimation and DMD.

The overall workflow for visualizing brain pulsatility involved four key steps: acquisition, image reconstruction, motion amplification and dynamic analysis (figure 1).

### 3.1. Acquisition

The 3D amplified MRI technique used balanced SSFP (vendor-specific name: FIESTA) as its base sequence. Dynamic signals were undersampled in Cartesian space with a variable density sampling scheme to provide a smooth transition from denser centre regions to sparser outer regions. A cardiac-gated acquisition was employed with peripheral cardiac gating with dynamic signals collected prospectively during free breathing across multiple heartbeats, and then retrospectively reconstructed to form an average heartbeat cycle. For an acceleration factor of 8, whole-head coverage at 1 mm isotropic resolution was achieved within 2 min, yielding a matrix size of 256 × 256 and a temporal resolution of approximately 50 ms, depending on HR. Imaging parameters for each use case are detailed in table 1.

**Table 1.** Demographics of scan participants. Imaging parameters in order are slice thickness/TR/flip angle/views per segment/FOV/temporal resolution. The frequency-encoding direction for all scans was in the superior–inferior direction.

use case	condition	age	sex	avg HR	imaging parameters
change in HR	resting	21	M	64	1.3 mm/2.8 s/25°/21 vps/ 25 cm/63 ms
	exertion			83	
elevated ICP	pre-LP	50	F	84	1.0 mm/3.1 s/25°/20 vps/ 22 cm/63 ms
	post-LP			86	
mTBI	pre-injury	16	M	62	1.3 mm/2.8 s/25°/19 vps/ 25 cm/63 ms
	post-injury			53	
test–retest	test	41	M	70	1.3 mm/2.8 s/25°/19 vps/ 25 cm/63 ms
	retest			66	

FOV, field of view; TR, repetition time.

### 3.2. Image reconstruction

Dynamic images were reconstructed from undersampled k-space data using specialized compressed sensing methods [28]. The k-adaptive-t autocalibrating method (kat ARC) was applied [29], leveraging both spatial and temporal correlations to recover missing data from the inherent redundancy in the dynamic signals.

### 3.3. Motion estimation

To reduce artefacts caused by pulsation in regions outside the brain, such as the mouth and nose, brain extraction was performed. Pulsatile motion in the cine images is subvoxel in nature with temporal fluctuations across cardiac phases containing subtle motion information. During amplification, images from each cardiac phase were spatially decomposed, and each component was temporally band-pass filtered to enhance the desired frequency range. Amplified components were recombined to produce the output cine images, with separate processing paths for visualization and quantification. For quantification, voxel displacement fields were calculated by solving a least-squares optimization problem to align spatial phase derivatives with temporal changes, then mapped back to the original unamplified images using cubic interpolation. It is important to note that the amplified cine images represent the underlying spatio-temporal variation in the motion patterns and not the actual motion as produced by registration-based methods.

Motion estimation was conducted under two frequency settings: a narrow band of ±0.2 Hz around the average recorded HR and a wideband of 0.1−4 Hz. Frequencies outside the selected range were attenuated, and results from both bands were reported.

### 3.4. Dynamic analysis

Dynamic changes in signal intensities and motion estimates were evaluated using three methods:

(i) Temporal signal changes along a line drawn through each image were visualized for both unamplified and amplified images to assess temporal variations aligned with cardiac phases.
(ii) Average signal intensity changes over time were calculated across the entire brain region, excluding non-brain areas such as the skull and surrounding tissues.
(iii) DMD [23,30,31] was applied to decompose the amplified cine data into a set of coherent spatial modes, scalar amplitudes and temporal frequencies. DMD allows high-dimensional datasets to be reduced into fundamental modes that characterize the system’s behaviour. Briefly, for a dynamical system X with *n* entries, the system has ‘*m*’ temporal snapshots with *n* >> m. Then, DMD decoposes the system into a spatial mode ‘Φ_M_’, scalar amplitude ‘*a*_M_’ and temporal component. ‘λ_M_’ contains real and imaginary (the frequency) components. Here, an optimized DMD model was used, centring data by the mean and employing 20 temporal snapshots. The resulting modes, frequencies and amplitudes were used to analyse changes in pulsatility as follows:

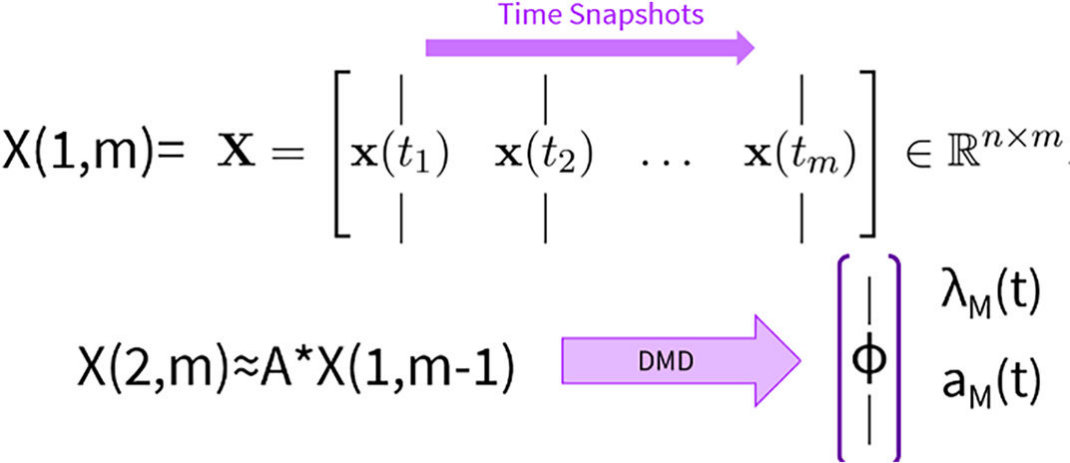


## 4. Results

**Figure 2.**
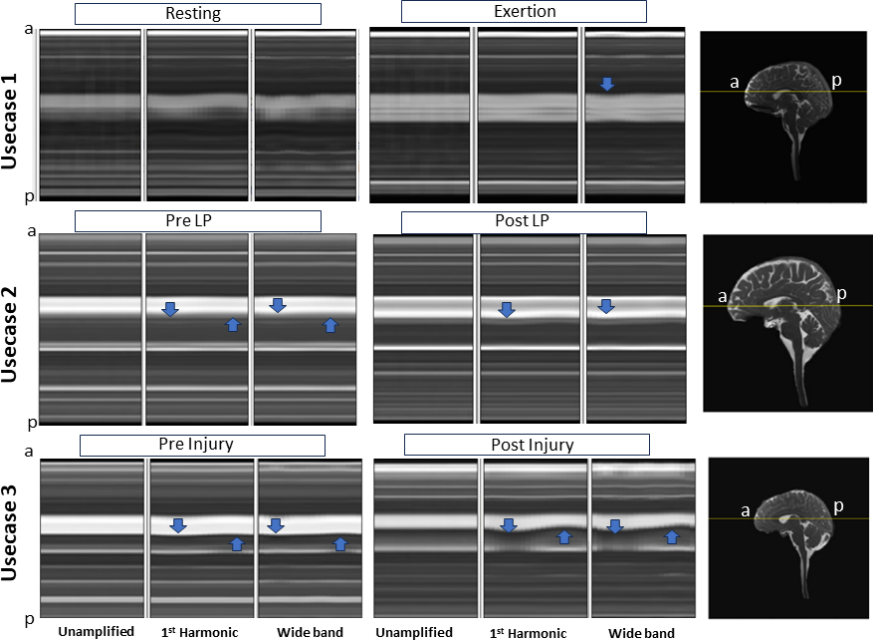
Temporal line profiles of original and amplified signals across different use cases. Each plot shows a posterior–anterior line through the lateral ventricle, with the *x*-axis representing one average cardiac beat and the *y*-axis representing a line in the image. The first row compares rest and elevated HR; the second row compares pre- and post-LP; and the third row compares pre- and post-mTBI. The first column displays unamplified signals, the second column shows results from amplifying the first harmonic and the third column displays the wideband amplification.

**Figure 3.**
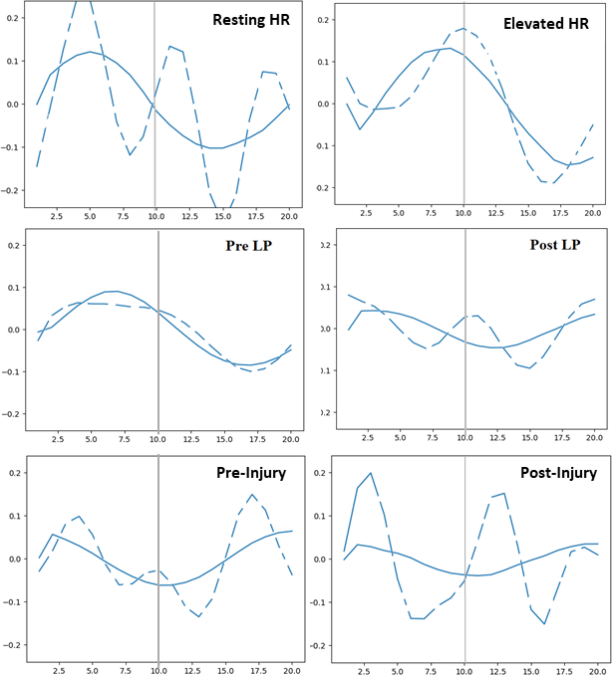
Normalized temporal variation in whole-brain signal intensities across different conditions. The first row shows resting HR versus elevated HR; the second row shows pre- and post-LP; and the third row shows baseline versus post-injury. The solid line represents the signal from the first harmonic amplification, while the dashed line represents the wideband amplified signal.

**Figure 4.**
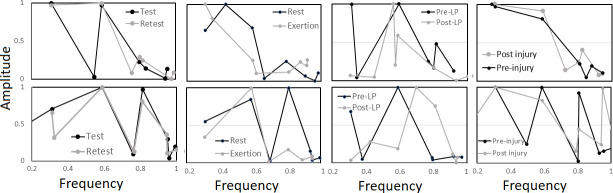
Frequency versus amplitude for different use cases. The top row displays results from first harmonic amplification, while the bottom row shows results from wideband harmonic amplification.

**Figure 5.**
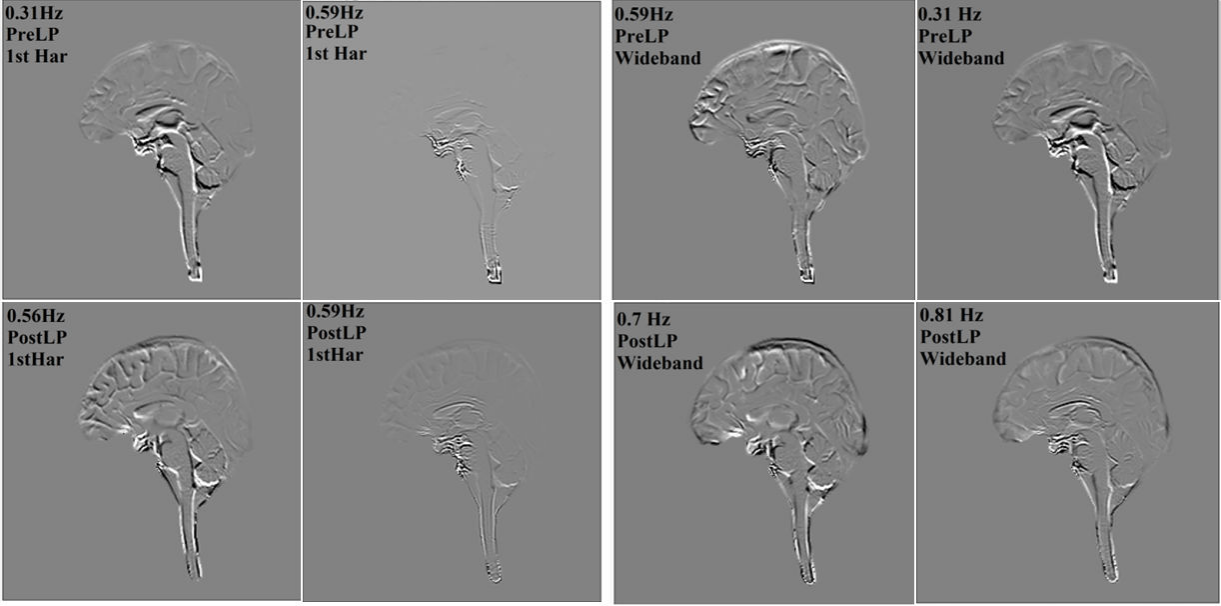
Spatial visualization of the DMD modes for the pre- and post-LP use case. The top row shows the top two modes for the pre-LP case, while the bottom row displays the top modes for the post-LP case.

**Figure 6.**
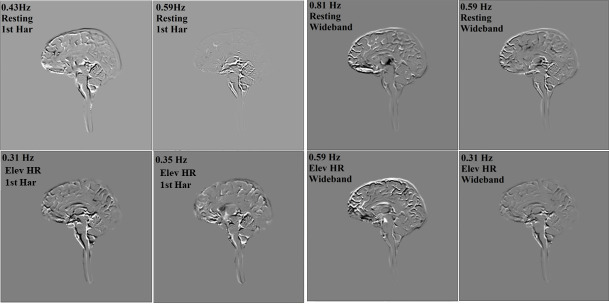
Spatial visualization of the DMD modes for rest and exertion use case. Top row shows the top two modes for the resting HR and the bottom row modes for the elevated HR.

Figures 2–6 and tables 2 and 3 present the findings from the dynamic analysis.

**Table 2.** DMD results from first harmonic amplification. Each mode’s frequency component and normalized amplitude are displayed with the amplitude of each mode indicated in parentheses. Only the modes up to the fourth largest amplitude are shown.

1st harmonic	resting HR	elevated HR	pre-LP	post-LP	pre-injury	post-injury	test	retest
mode 1	0.43 (1.0)	0.31 (1.0)	0.595 (1.0)	0.563 (1.0)	0.29 (1.0)	0.31 (1.0)	0.31 (1.0)	0.59 (1.0)
mode 2	0.59 (0.69)	0.35 (0.82)	0.32 (0.99)	0.59 (0.59)	0.31 (0.97)	0.594 (0.91)	0.59 (0.97)	0.31 (0.97)
mode 3	0.31 (0.66)	0.92 (0.28)	0.815 (0.48)	0.575 (0.21)	0.588 (0.81)	0.83 (0.4)	0.8 (0.23)	0.8 (0.29)
mode 4	0.79 (0.26)	0.59 (0.27)	0.77 (0.25)	0.8 (0.172)	0.81 (0.22)	0.8 (0.25)	0.84 (0.15)	0.82 (0.25)

**Table 3.** DMD results from wideband amplification. Each mode’s frequency component and normalized amplitude are displayed with the amplitude of each mode indicated in parentheses. Only the largest four modes are shown.

wideband	resting HR	elevated HR	pre-LP	post-LP	pre-injury	post-injury	test	retest
mode 1	0.81 (1.0)	0.59 (1.0)	0.59 (1.0)	0.7 (1.0)	0.31 (1.0)	0.31 (1.0)	0.59 (1.0)	0.59 (1.0)
mode 2	0.59 (0.85)	0.31 (0.35)	0.31 (0.68)	0.81 (0.76)	0.59 (0.99)	0.95 (0.98)	0.81 (0.97)	0.81 (0.81)
mode 3	0.31 (0.55)	0.81 (0.18)	0.92 (0.09)	0.43 (0.28)	0.81 (0.92)	0.59 (0.83)	0.31 (0.71)	0.31 (0.66)
mode 4	0.94 (0.16)	0.95 (0.1)	0.97 (0.08)	0.59 (0.19)	0.5 (0.25)	0.17 (0.57)	0.13 (0.44)	0.95 (0.36)

### 4.1. Temporal line profiles

Figure 2 shows the temporal line profiles comparing unamplified and amplified signal intensities along an anterior–posterior line through the lateral ventricle on a mid-sagittal plane. Signals were extracted for each of the 20 cardiac phases and plotted as line profiles. Deviations from a straight horizontal line (representing steady state) indicate dynamic motion; the more pronounced the deviation, the greater the motion. Both first harmonic and wideband amplification results are shown. Subtle temporal changes in signal intensity are visible in the unamplified signal, while the amplified signals show noticeable rhythmic changes, as indicated by the arrows.

In the elevated HR use case, the temporal variations in the line profile were greater at rest than during elevated HR, suggesting that increased HR impacts brain pulsatile motion.

In the elevated ICP use case, the line profile reveals reduced pulsatility in both pre- and post-LP. Subtle differences in the location and nature of the peaks in the first harmonic and wideband cases are marked by arrows.

In the mTBI use case, figure 2 shows similar pulsatility patterns for both the first harmonic and wideband methods with qualitative differences. Post-injury pulsatility appears greater than pre-injury within the selected line profile, as indicated by the arrows. Unlike the multiple peaks observed in the elevated HR use case, the mTBI line profile shows distinct systolic and diastolic peaks, which were only observed in the wideband result.

### 4.2. Whole-brain average signal intensity

Figure 3 shows the average signal intensity for the whole brain across the 20 cardiac phases, plotted to display the overall temporal changes following amplification. In each use case, the first harmonic results reveal near-cyclical behaviour with a distinct peak in signal intensity. The timing of this peak within the cardiac cycle varies depending on the use case. In contrast, the wideband results are also pulsatile but display multiple peaks within a single cardiac cycle. In the elevated ICP case, the solid line shows that the systolic and diastolic peaks differ between pre- and post-LP conditions with smaller overall signal intensity changes in both states, ranging from −0.1 to 0.1. Notably, in the wideband analysis, pre-LP brain pulsatile motion is not influenced by other frequencies, while post-LP results show the hallmark multiple peaks of a wideband signal.

### 4.3. Dynamic mode decomposition analysis

**Figure 7.**
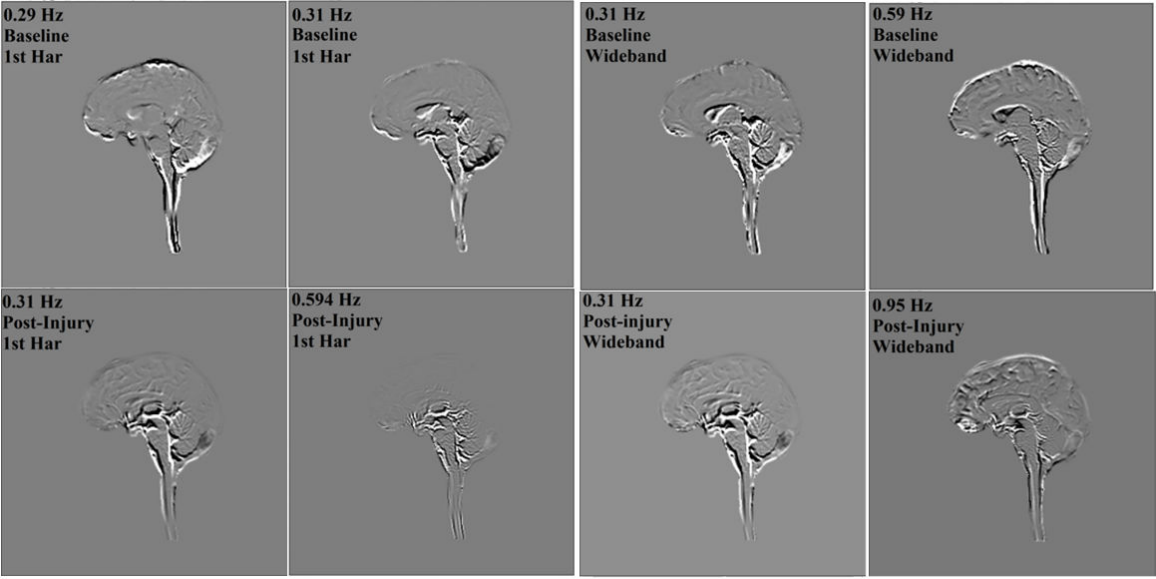
Spatial visualization of the DMD modes for the mTBI use case. The top row shows the top two modes for the baseline state, while the bottom row displays the top modes for the mTBI state.

Figures 4–7 and tables 2 and 3 summarize the results of DMD analysis. Figure 4 shows frequency versus amplitude for each use case derived from the DMD modes. Modes are arranged from lowest to highest frequency with each pair of subsequent modes connected by a line segment. In general, frequencies were in the range of 0.2–1 Hz. Mode amplitudes were normalized, with the dominant mode set to an amplitude of 1.0. The test–retest results indicate that dominant modes are generally preserved across tests with the wideband analysis showing a high-frequency mode around 0.8 Hz as dominant.

In the elevated HR use case, the wideband results showed the removal of the 0.8 Hz mode. Significant differences in dominant frequencies were observed between pre- and post-LP conditions.

DMD analysis for the first harmonic across the whole brain highlighted a prominent observation in the mTBI use case: post-injury, a high-amplitude component at 0.83 Hz emerged, while the pre-injury state showed moderate amplitude at 0.81 Hz (table 2). This suggests a slight shift in pulsatility post-injury. Tables 2 and 3 also show similar frequencies for both first harmonic and wideband analyses in pre- and post-mTBI, with post-injury amplitudes slightly higher than pre-injury in the first harmonic results. An example of this injury-related increase in amplitude is shown in figure 4.

### 4.4. Frequency and amplitude analysis by dynamic mode decomposition modes

Table 2 presents DMD mode frequencies and amplitudes from the first harmonic amplification. The DMD modes typically displayed frequencies between 0 and 1 Hz, with only the top four modes (those with the highest amplitudes) shown for simplicity. The mode with the maximum amplitude is assigned 1.0, followed by other modes ranked accordingly.

In the resting condition, the mode at 0.43 Hz had the highest amplitude, followed by 0.59 and 0.31 Hz modes. The mode at 0.59 Hz, prominent at rest, was not among the higher amplitude modes during elevated HR, indicating that exertion alters the dominant brain motion mode. In the wideband result, exertion showed dominant motion within a narrow frequency range with 0.59 Hz having the maximum amplitude, consistent with the temporal changes in overall signal intensity shown in figure 3.

### 4.5. Elevated intracranial pressure use case

For the elevated ICP use case, the first harmonic results showed that 0.32 and 0.59 Hz modes had the largest amplitudes with a higher frequency component around 0.8 Hz present but with lower amplitude. Notably, at lower ICP, motion was primarily within the 0.56–0.59 Hz range. In the wideband results, 0.59 Hz was the dominant mode pre-LP, followed by 0.31 Hz, while post-LP, the dominant frequencies shifted to higher frequencies of 0.7 and 0.8 Hz.

### 4.6. Spatial visualization of motion

A mid-sagittal slice was selected to visualize regions of motion across different conditions. In the pre-LP state, the 0.32 Hz mode showed prominent motion in the lateral ventricles, midbrain, brainstem, fourth ventricle and cerebellar tonsil. Post-LP, motion was visible within the brain parenchyma with reduced motion along the edges of the lateral ventricles.

In the resting state, the 0.43 Hz mode showed motion primarily in the midbrain and brain parenchyma, while during exertion, motion was distributed between the 0.31 and 0.35 Hz modes.

In the mTBI case, baseline motion was observed at 0.29–0.31 Hz, while post-injury, motion was present at 0.31 and 0.59 Hz – but the latter with a slight reduction in amplitude. In the wideband analysis, post-injury motion spanned a broader range, from 0.31 to 0.95 Hz.

## 5. Discussion

In this study, we visualized the pulsatile motion of brain tissue using cine MRI images, retrospectively cardiac-gated to represent average motion over a single cardiac cycle. The motion, revealed through specialized post-processing, demonstrated synchronized movement within the brain. A key contribution of this work is the application of DMD to provide a spatio-temporal analysis of the pulsatility. Unlike previous studies that described motion using difference maps or cinematic movies of pulsatility, the current study utilizes DMD to capture both dominant spatial and temporal variations, offering an alternative method for describing amplified motion.

Dynamic images show intensity changes throughout the cardiac cycle. These changes are expected to vary in response to alterations in the underlying physiological processes. This work demonstrates that temporal motion can be decomposed into dominant modes corresponding to the different motion characteristics. Notably, in most cases, two dominant dynamic modes (by amplitude) were sufficient to represent motion in the first harmonic results, with additional modes typically exhibiting less than 50% of the maximum amplitude. When visualized, these lesser modes contributed minimal motion, hence were not plotted further. For example, in the elevated ICP use case, only the dominant mode displayed a significant motion pattern, as shown in figure 5.

The use of aMRI to study pulsatile motion changes provides a novel approach to understanding mTBI mechanisms. In moderate to severe TBI, structural changes such as contusion, haemorrhage and oedema are often visible on conventional imaging (e.g. T1w, T2w, FLAIR). However, a distinguishing feature of mTBI is the absence of these overt pathoanatomic findings [32,33]. In milder injuries, subtle microswelling is diffusely distributed throughout the brain. mTBI-related microswelling and its impact on subvoxel pulsatile motion could be detected using aMRI, which probably manifested spatially as vastly reduced ventricular motion post-injury. Strained neural tissue fibres and autonomic decoupling could be additional explanatory variables contributing to altered pulsatile motion post-mTBI [34]. The dynamic analysis here indicates that this approach to motion estimation holds promise for detecting subtle injury-associated alterations in pulsation secondary to mild forms of brain trauma.

In idiopathic intracranial hypertension, CSF homeostasis is thought to elevate ICP and potentially increase overall CSF volume. While various neuroimaging patterns have been proposed for recognizing elevated ICP, none are universally diagnostic. Imaging the brain’s pulsatile motion offers an opportunity to develop new biomarkers with the hypothesis that elevated ICP may uniquely alter pulsatility. In the use case here, we observed that the motion was reduced overall pre-LP and the dominant mode was altered post-LP. A primary challenge has been interpreting variations in motion, including both inter- and intrasubject differences. At this stage, we show that elevated ICP does exhibit distinct pulsatile motion characteristics in a detectable fashion. Larger cohort studies are needed to identify and study such changes. For ICP use case, results interestingly suggest differences in modal frequencies associated with motion, which vary depending on the chosen attenuation band during motion estimation warranting the use of both bands for interpretation.

In general for all use cases, we notice that wideband results showed better diagnostic ability in detecting differences between the two states chosen.

When interpreting motion using modes and frequencies, two assumptions should be noted. First, the discrete nature of the input data: the cine input to DMD is binned into 20 cardiac frames, resulting in a temporal resolution of approximately 0.05 Hz for a HR of 60 beats per minute. The observed signal variations were smooth, so mode frequencies differing by less than this sampling window could theoretically be linearly combined. For example, in the post-injury use case, modes at 0.83 and 0.8 Hz were measured with different amplitudes. Second, the time range for DMD was normalized from 0 to 1 across all use cases. Although investigating the influence of HR on motion is beyond the scope of this study, this normalization approach provides consistency across analyses.

Finally, the physiological effects of elevated HR vary depending on the underlying cause, such as exercise, caffeine intake or stress. These changes, along with the shorter duration of each cardiac cycle, may disrupt the laminar flow of blood and CSF, especially in complex areas like the brainstem and hence reduce overall intracranial pulsatility. The elevated HR was straightforward to interpret as the stimulus was controlled using pulsatility of arterial input via overall HR change. We recommend this use case, particularly for newly developed techniques investigating pulsatility.

### 5.1. Limitations

Several limitations must be considered. First, the sample size is limited to four participants, which restricts the generalizability of the findings. Future studies with larger and more diverse cohorts are needed to confirm these observations and assess intersubject variability in pulsatile motion across different populations.

Second, the retrospective cardiac gating method used in this study produces averaged motion data, which may not fully capture the nuances of beat-to-beat variability in brain pulsatility. Real-time or prospective cardiac gating may enhance temporal accuracy and allow for more precise tracking of physiological fluctuations across individual heartbeats.

Additionally, the current study employed a nominal HR assumption, which standardizes data for analysis but may not fully reflect individual physiological differences in HR variability. Although this normalization provides consistency across cases, it may obscure frequency shifts linked to true HR changes.

Finally, aMRI relies on subvoxel motion detection, which is subject to potential errors in spatial resolution and motion estimation. Artefacts from extracranial sources, such as mouth and nasal cavity motion, may also interfere with brain motion measurements, despite brain extraction processes. Advanced artefact-reduction techniques or segmentation methods may be necessary to improve accuracy in clinical settings.

## 6. Conclusion

This study demonstrates the potential of aMRI with DMD to visualize brain pulsatility, providing insights into cerebral biomechanics in conditions such as mTBI and elevated ICP. By visualizing dominant spatial and temporal variations in brain tissue motion, this approach offers a new method to compare and contrast subtle physiological changes that may serve as biomarkers for injury and pressure-related pathologies. As we expand our use cases in future to study larger cohorts and also other pathologies, we believe that each pathology may reveal a unique motion characteristic signature to the underlying pathophysiology. We also noticed that using the wider attenuation band shows better prospects in its ability to detect differences in underlying pulsatility.

## Data Availability

In line with the approved ethics documentation from this study, endorsed by the New Zealand Health and Disability Ethics Committees (NZ HDEC), and in accordance with our indigenous and community engagement policies, non-identifiable data are available upon request and subsequent approval by Mātai Ngā Māngai Māori Board (contacted at nmm@matai.org.nz). This protocol ensures adherence to ethical standards, respects community involvement and upholds data sovereignty principles.
